# Untangling Discrimination: The Impact of Internalizing Oppression on HIV Treatment Engagement Among Young Black Sexual Minority Men in the Southern US

**DOI:** 10.1007/s10461-025-04774-z

**Published:** 2025-06-05

**Authors:** Carrie L. Nacht, Cody Lentz, Wilson Vincent, Daniel Siconolfi, Lance M. Pollack, Susan M. Kegeles, Chadwick K. Campbell, Adedotun Ogunbajo, Erik D. Storholm

**Affiliations:** 1 School of Public Health, San Diego State University, San Diego, CA, USA; 2 Herbert Wertheim School of Public Health and Human Longevity Science, University of California San Diego, San Diego, CA, USA; 3 Department of Psychology & Neuroscience, Temple University, Philadelphia, PA, USA; 4 RAND, Behavioral and Policy Sciences, Santa Monica, CA, USA; 5 Division of Prevention Science, Department of Medicine, University of California San Francisco, San Francisco, CA, USA; 6 Department of Family Medicine, Center for HIV Identification, Prevention and Treatment Services, University of California Los Angeles, Los Angeles, CA, USA

**Keywords:** Sexual minority men, Antiretroviral therapy, Highly active, HIV, Minority stress theory, Social stigma

## Abstract

Black sexual minority men living with HIV (BSMM+) in the Southern United States encounter multiple forms of discrimination, which are associated with decreased HIV care engagement. We analyzed data from 166 BSMM + in the South to assess direct associations between experiences of racism, heterosexism, and HIV-related discrimination with HIV care engagement. We further investigated indirect associations through three mediators: internalized racism, internalized heterosexism, and internalized HIV stigma. Experienced heterosexism was indirectly associated with HIV care engagement through internalized HIV stigma (*b*_indirect_ = −0.039, *p* = 0.098). HIV discrimination was associated with internalized heterosexism (*b*_direct_ = 0.577, *p* = 0.007) and internalized racism (*b*_direct_ = 0.253, *p* = 0.009). There were significant direct associations between internalized HIV stigma and HIV care engagement (*b*_direct_ = −0.040, *p* = 0.001) and experienced heterosexism and internalized HIV stigma (*b*_direct_ = 0.974, *p* = 0.050). These findings aim to understand the factors affecting HIV care engagement among this population to improve HIV care engagement.

## Introduction

Black sexual minority men (BSMM) in the United States (US) are disproportionately impacted by HIV; in 2019, BSMM comprised 26% of all new HIV diagnoses [1]. Further, BSMM living with HIV (BSMM+) have poorer outcomes across all steps of the HIV care continuum: HIV diagnosis, linkage to HIV care, retention in HIV care, initiating antiretroviral therapy (ART), and achieving and maintaining HIV viral load suppression [2, 3]. BSMM + are less likely to be aware of their serostatus compared to White SMM+ [4], and undiagnosed infection increases the likelihood of unknowingly transmitting HIV to others [5]. Further, compared to other racial/ethnic groups, BSMM + are less likely to be linked to HIV care after diagnosis [6, 7], be retained in care [8], use or adhere to antiretroviral therapy [9–14], and achieve viral suppression [6, 15–17].

In 2019, the US South accounted for more new HIV cases among SMM than any other region in the US (51%), with nearly twice as many new diagnoses occurring in BSMM compared to White and Hispanic/Latino SMM [1]. People living with HIV (PLWH) in the South are more likely to be unaware that they are living with HIV [18] and are less likely to receive timely medical care or treatment [19]. Despite the disproportionate risk for suboptimal HIV outcomes in the US South, this area has been largely under-studied and remains a focus in the CDC’s Ending the HIV Epidemic (EHE) initiative [20].

**Fig. 1:**
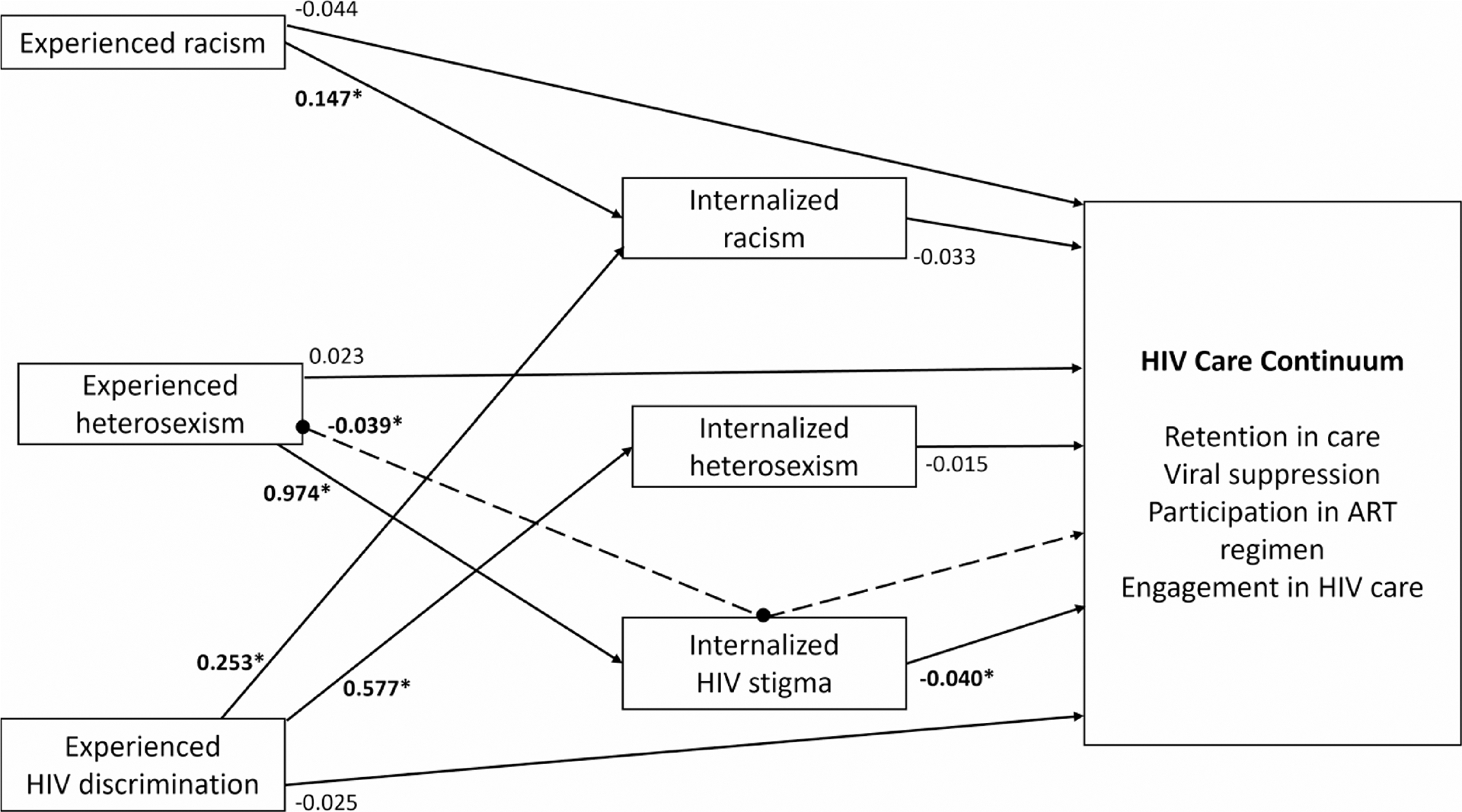
Structural model HIV care continuum. *Denotes significant association. Dashed line indicates indirect path

Identity-related discrimination—negative experiences related to one’s gender, sexual orientation, or racial identities– likely contributes to the HIV care continuum disparities observed among BSMM+. As suggested by minority stress theory, minoritized populations often experience increased stress resulting from experienced and internalized stigma, prejudice, and discrimination related to their socially marginalized identities [21–24]. Minority stress theory further outlines a potential pathway through which identity-related discrimination could contribute to known health disparities, such as reduced engagement in the HIV care continuum [21–24]. The theory contends that minoritized individuals first experience objectively distressing distal stressors, namely, identity-related acts of discrimination, rejection, or violence by others. Second, the identityrelated messages conveyed through repeated exposure to these distal stressors may become internalized within the individual (e.g., internalized oppression) [25, 26]. In other words, these stressors become more proximal as the individual internally processes and attempts to cope with the stigmatizing messages to which they are exposed (Fig. 1) [21]. This process of internalization– or integrating social messaging into one’s conception of the self [27]– can lead to adverse mental health outcomes among BSMM+ [28].

In addition to minority stress theory, this analysis utilizes the intersectionality theoretical framework. Intersectionality theory posits that identities are not independent, and that the nexus of different social positions jointly contributes to health and life experiences [29–31]. Intersectional discrimination, or policies, practices, and behaviors that perpetuate inequities based on intersecting marginalized social identities and positions [32–34], has been associated with lessthan-optimal engagement in the HIV care continuum. This is particularly relevant for BSMM+, as they encounter stereotypes related to multiple marginalized statuses related to sexuality, race, and HIV status [35, 36]. Previous research has shown that BSMM + are likely to encounter discrimination because they exist at the intersection of multiple stigmatized identities related to race, sexuality, and HIV status [21, 37]. Specifically, previous research has shown that experiences of discrimination of HIV status, racial identity, and sexual orientation among BSMM + are associated with reduced odds of accessing HIV care services [38], a greater likelihood of exiting HIV care.

Despite the marked HIV-related disparities observed among BSMM + in the South, few studies have investigated the relationship between multiple forms of stigma and engagement in the HIV care continuum in this group [39–41]. The present work aims to address these gaps in the literature by investigating cross-sectional associations between distinct forms of discrimination (based on race, HIV-positive status, and non-heterosexual orientation), internalized stigma, and HIV care engagement among a cohort of BSMM + in the US South.

## Methods

### Parent Study Participant Recruitment and Study Design

This analysis presents data from the fourth assessment time point in a longitudinal cohort study of BSMM + recruited from Dallas and Houston, Texas [42]. Recruitment methods have been published in publicly available papers [43, 44]. Briefly, baseline recruitment occurred between September 2015 and November 2016 using a long chain peer referral recruitment strategy that was implemented based on respondent-driven sampling methods [45–47]. Two organizations identified BSMM + through flyers, referrals from community-based HIV organizations, word of mouth, and at venues frequented by this population (e.g., bars, clubs). Once recruited, participants became “seeds,” who then recruited up to 12 other BSMM + into the study and received $15 for each participant recruited. At the time of initial recruitment, eligible participants had to be 18–29 years of age, live in the greater Dallas or Houston metropolitan areas, and be living with HIV based on rapid HIV tests conducted before the administration of the baseline survey. Thus, this is a community-based sample of men living with HIV, and participants were not recruited at medical centers providing treatment. The current study uses data from Time 4 (2022).

### Measures

#### Sociodemographic Characteristics

Age, race/ethnicity, sex assigned at birth, gender, sexual orientation, income in the past year, highest level of education, employment status, health insurance, and housing in the past year were self-reported by participants. All were collected at Time 4, except race, ethnicity, and sex assigned at birth, which were collected during the Time 1 visit (2015–2016).

#### Experienced Racism, Heterosexism, and HIV Discrimination

Experienced racism, heterosexism, and discrimination based on HIV/AIDS status were self-reported using 10 items from Bogart et al.’s Multiple Discrimination Scale [48]. Participants were asked if they experienced 10 negative events in the past year (e.g., “*Treated with hostility/coldness by strangers”*, *“Denied a job/lost a job”)*. Response options were (1) yes, more than once, (2) yes, once, and (3) no. If participants selected options (1) or (2), they were asked to identify if they felt this experience was due to their race/ethnicity, HIV status, sexual orientation, or some other reason (*“Of the events that happened to you in the past year*, *was it based on your…”*). The number of discrimination events were summed, where a higher score reflects greater experienced discrimination.

#### Internalized Racism

Internalized racism was self-reported using items from the Cross Ethnic-Racial Identity Scale-Adult (CERIS-A) [49]. The self-hatred scale includes four items asking about internalized negative feelings due to the participant’s ethnic/racial background, such as “*When I look in the mirror*, *sometimes I do not feel good about the ethnic/racial group I belong to”* and “*Privately*, *I sometimes have negative feelings about being a member of my ethnic/racial group”*. For each item, participants endorsed one of seven Likert-type response options ranging from strongly disagree (1) to strongly agree (7). Items were averaged for a final score, where a higher score reflects greater internalized racism (α = 0.91).

#### Internalized Heterosexism

Internalized heterosexism was self-reported using an adapted and expanded version of a scale containing items about guilt and disliking oneself for liking men [50] that has been used and described previously [51]. This expanded scale included six questions inquiring about internalized negative feelings associated with one’s sexual identity, such as “*Do you ever wish that you were attracted only to women?*” and “*How happy are you being gay or bisexual?”*. For each item, participants endorsed one of five Likert-type response options ranging from not at all (1) to a great deal (5). Items were summed for a final score, where a higher score reflects greater internalized heterosexism (α = 0.80).

#### Internalized HIV Stigma

Internalized HIV stigma was self-reported using an adapted version of the Internalized AIDS-Related Stigma Scale (IA-RSS) [52], which has been used and described elsewhere [53]. This scale has six items that inquire about one’s beliefs and behaviors about one’s HIV status, such as “*To what extent does being HIV-positive make you feel dirty?*” and “*How difficult is it for you to tell people about your HIV infection?”*. Participants selected a response from a five-item Likert scale, with options ranging from not at all (1), to extremely (5). Items were summed for a final score, where a higher score reflects greater internalized HIV stigma (α = 0.92).

#### HIV Care Continuum Engagement

The outcome in this analysis was *HIV care continuum engagement*, calculated as a summed composite variable (range 0–4) from the following areas: engagement in HIV care, retention in care, participation in an ART regimen, and viral suppression [54]. Engagement in HIV care was assessed using the question, *“In the past 12 months*, *how many times have you received care for HIV? This can include video or phone visits but does not include care received in an ER or during an in-patient hospital stay*,*”* where a response greater than 0 was considered active engagement in HIV care (a value of 1). Retention in care was assessed with the question “*When was the last time you had a viral load test?*”, where a response within the last 6 months was considered retained in care (a value of 1). Participation in an ART regimen was assessed using the question *“Are you currently taking HIV medications (usually called “ART” or “antiretroviral therapy”)? This does not include nutritional supplements*,*”* where an affirmative response was considered active in an ART regimen (a value of 1). Viral load was assessed using the question “*What was the result of your-most recentviral load test?*”, where a response of undetectable was considered virally suppressed (a value of 1). All measures were self-reported.

### Data Analysis

Univariate descriptive analysis was performed in SAS, version 9.4 (SAS Institute Inc., Cary, North Carolina). Tests of mediation using regression [55, 56] were conducted in M*plus* version 8 [57], which provides state-of-the-art missing data handling options. We tested the extent to which experienced racism, experienced heterosexism, and experienced HIV discrimination were associated with engagement in the HIV care continuum directly or indirectly via internalized racism, internalized heterosexism, and internalized HIV stigma. Specifically, three direct paths were tested, one for each predictor (experienced racism, experienced heterosexism, and experienced HIV discrimination) to the HIV care continuum outcome. Nine indirect paths were tested, one for each predictor through each form of internalized stigma (internalized racism, internalized heterosexism, and internalized HIV stigma) to the HIV care continuum outcome. The HIV care continuum engagement dependent variable was treated as ordinal across five levels. Thus, given an ordinal outcome variable, a weighted least squares with mean and variance-adjusted test statistics (WLSMV in M*plus*) was used. As this was an exploratory analysis, α = 0.10 was used. Multiple imputation was performed to handle missing data. Unstandardized regression coefficients (*b*), standard errors (*SE*), 90% confidence intervals (CI._90_), and *p*-values are reported in the text and tables. Standardized regression coefficients (b*) are reported in the tables. As standardized coefficients are not typically produced in analyses of indirect effects in M*plus*, the Sobel method was used to provide an approximate value of standardized indirect effects to facilitate interpretation. The model was tested for the presence of collinearity in Stata/MP version 18 [58] with tolerance values derived using ordinal probit regression. This approach utilizes the probit function, comparable to the regression coefficients produced using WLSMV in M*plus*. Tolerance values of 0.10 or greater indicate that collinearity was detected in the model.

## Results

### Participant Characteristics

Of the 172 participants who completed the Time 4 survey, a substantial majority self-reported being: Black or African American (95.4%), non-Hispanic/Latinx (97.6%), assigned male sex at birth (98.2%), and homosexual/gay (80.2%) (Table 1). This cohort was relatively socioeconomically disadvantaged, with most participants (63.4%) reporting an annual income less than $40,000 and almost half (40.7%) having earned a high school degree or less. More than a quarter of this cohort reported having no health insurance (29.1%) and being unemployed or on disability (29.1%). About 1 in 6 (17.4%) reported having unstable housing in the past year.

Six participants (3.5%) had missing data for all variables of interest and were dropped from the analysis. Thus, the final analytic sample included 166 participants.

### Regression Analysis

The observed tolerance values ranged from 0.054 to < 0.001 from the first iteration to the fourth (i.e., the last) in ordinal regression upon model conversion. As all values were less than 0.10, no collinearity was detected in the model. The results from the final model can be found in Table 2. There was a statistically significant indirect inverse association from experienced heterosexism to engagement in the HIV care continuum via internalized HIV stigma (*b*_indirect_ = −0.039, *SE* = 0.023, CI._90_ = [−0.077, −0.001], *p* = 0.098). The constituent direct association between internalized HIV stigma and engagement in the HIV care continuum was statistically significant (*b*_direct_ = −0.040, *SE* = 0.012, CI._90_ = [−0.061, −0.020], *p* = 0.001). Additionally, the constituent direct association between experienced heterosexism and internalized HIV stigma (*b*_direct_ = 0.974, *SE* = 0.497, CI._90_ = [0.156, 1.792], *p* = 0.050) was statistically significant. Notably, no other indirect paths achieved statistical significance.

There were several other significant direct associations in the model while controlling for all variables simultaneously. The constituent direct association between experienced racism and internalized racism was statistically significant (*b*_direct_ = 0.147, *SE* = 0.085, CI._90_ = [0.035, 0.259], *p* = 0.033). Another statistically significant direct association was between experienced HIV discrimination on internalized racism (*b*_direct_ = 0.253, *SE* = 0.098, CI._90_ = [−0.036, 0.492], *p* = 0.009). The constituent direct association between experienced HIV discrimination and internalized heterosexism was also statistically significant (*b*_direct_ = 0.577, *SE* = 0.213, CI._90_ = [0.227, 0.927], *p* = 0.007). Also, the constituent direct association between gender and internalized HIV stigma (*b*_direct_ = 4.786, *SE* = 2.133, CI._90_ = [1.277, 8.295], *p* = 0.025). There was a significant inverse association between income and internalized racism (*b*_direct_ = −0.242, *SE* = 0.118, CI._90_ = [−0.436, −0.049], *p* = 0.039). Finally, there was a significant inverse association between age and engagement in the HIV care continuum (*b*_direct_ = −0.062, *SE* = 0.036, CI._90_ = [−0.122, −0.003], *p* = 0.085).

## Discussion

Informed by minority stress theory and an intersectionality framework, we aimed to understand the relationship between unique forms of discrimination, corresponding forms of internalized oppression, and engagement in the HIV care continuum among BSMM + in the US South. Overall, we found that participants who experienced heterosexism reported greater internalized HIV stigma and, in turn, significantly reduced engagement in the HIV care continuum. Our findings reveal a highly nuanced relationship between experienced discrimination and internalized stigma. Specifically, discrimination based on one identity (e.g., HIV status) may be significantly correlated with the internalization of other intersecting forms of oppression (e.g., racism). This underscores the critical need for taking an intersectional approach when addressing internalized oppression among BSMM+ [59, 60]. Recognizing that aspects of identity are not discrete but deeply interconnected, future approaches must be holistic and integrative to effectively support the well-being of BSMM+.

This relationship is not particularly surprising, in that it aligns with prevalent stereotypes that conflate gay or bisexual identity and same-sex behavior with HIV risk. Long-entrenched discriminatory stereotypes have posited that SMM are disproportionately burdened by HIV due to a high number of sexual partners [61]. This messaging may be especially prevalent in the US South, where oppressive anti-LGBTQ policies and some religious institutions and cultures may perpetuate homonegativity by associating homosexuality, sexually permissive norms, “sinfulness,” and HIV [62–66]. This may be particularly relevant for the Black community, the most religious racial or ethnic group in the US [67], where the church is a critical aspect of the community that acts as a source of social support, health services, and political and social movements [68, 69]. Thus, BSMM may face discrimination or ostracization from their church and community members when they reveal their sexual identity. Other studies have found that experiences of heterosexism were significantly associated with greater sexual risk behavior, HIV infection rates, and decreased engagement in the HIV care continuum, suggesting that it is the experience of discrimination and the internalization of oppression that likely contributes to HIV-related disparities among sexual minority populations [70–74]. As such, BSMM who attend churches that discourage homosexuality have been associated with negative outcomes related to HIV, such as HIV infection and HIV risk behaviors [75].

Previous studies have reported that internalized stigmas may mediate the relationship between experienced discrimination and the suboptimal outcomes often observed among minority populations [21, 76]. Being confronted with repeated discrimination based on race, sexual orientation, and HIV status has been shown to be associated with internalized stigma against that respective identity [24, 28, 77–80]. Fortunately, experienced discrimination may not always be internalized to the same degree. Protective factors such as resilience, social support, and adaptive coping strategies may decrease the burden of intersectional discrimination [81]. However, intersectionality research has suggested prioritizing the mutually reinforcing nature of different forms of oppression rather than individually studying each form separately [34, 82]. Thus, targeting internalized stigma may be one avenue for future interventions to facilitate greater acceptance and empowerment to mitigate the negative impact of experienced intersectional discrimination. An intervention among BSMM + that leveraged peer-led support networks was associated with decreased HIV stigma and improved status disclosure, HIV care adherence [83]. Adapting a similar intervention to BSMM + in the South specifically may be beneficial to mitigate the internalization of intersectional identity discrimination.

Of note, this population had relatively high levels of housing instability, substance and alcohol abuse, intimate partner violence (IPV), and depression compared to national averages [84]. Housing instability and drug use are associated with lower viral suppression in Black SMM compared to White SMM [85]. Experiencing IPV has also been found to predict poor HIV care engagement outcomes among a sample of PLWH with a large proportion of SMM [86]. Preliminary research among BSMM in the South has found that church-based interventions to mitigate some of these co-occurring epidemics have been deemed feasible, acceptable, and potentially successful avenues warranting further exploration [87–89]. Future research should continue to explore and refine these church-based models, ensuring that they are tailored to be maximally empowering, stigma-free, and effectively meet the needs of the BSMM + community. Collaborations with faith-based organizations that have a strong presence in the community can enhance trust and participation in these interventions. Additionally, incorporating feedback from BSMM in the design and implementation phases can ensure the interventions are responsive and relevant to their lived experiences.

In tandem with targeting internalized intersectional stigma, future interventions offered in community-based settings may benefit from leveraging the importance of community to improve HIV care. Community-based settings, such as community centers and local organizations that are maximally free of stigma and oppression, provide a trusted and familiar environment that can significantly enhance the acceptance and effectiveness of health interventions among BSMM+. Leveraging these settings can create a holistic intervention approach that addresses both the medical and social needs of this population by targeting internalized oppression, fostering self-acceptance, rejecting negative stereotypes, and helping to build pride. Critical consciousness-based interventions may be especially well-suited to address multiple simultaneous forms of oppression and their negative effects on wellbeing among BSMM [67]. By embedding interventions within community-based settings that are LGBTQ-affirming and HIV stigma-free, we can take advantage of existing support systems that are crucial for fostering engagement and retention in HIV care. These environments can offer a sense of belonging and security, which is essential for BSMM + who may feel marginalized or stigmatized in traditional healthcare settings.

Community mobilization is an important HIV prevention intervention focused at the community level, exemplified by targeted interventions such as the Connect to Protect project, which aims to reduce HIV incidence by altering structural elements in urban communities [90]. It is particularly promising for BSMM, a group for whom community mobilization addresses critical social factors such as social isolation [91]. By integrating technology-based interventions, projects like the POSSE intervention, which specifically tailored strategies for the House Ball Community to reduce multiple sexual partners and condomless intercourse [92]. Additionally, the mPowerment project has shown success in fostering community engagement and promoting safe sexual practices among BSMM through peer-led initiatives and social marketing campaigns [51]. Successful community mobilization strategies ensure that interventions are culturally sensitive and effectively engage the target demographic. By fostering inclusive interventions that encourage education, dialogue, and resource access, community mobilization can help to combat stigma and empower communities to enact sustainable changes to improve HIV care continuum outcomes for BSMM living with HIV.

While this analysis provided valuable insights, there were some limitations that should be noted. First, the analyses were cross-sectional; as such, the present findings should not be used to form causal inferences. Future longitudinal research could provide deeper insights into causal relationships. Second, the sampled cohort included BSMM + living in the US South, which offers valuable region-specific insights but may limit the generalizability to BSMM + in other geographic regions. Expanding this research to include diverse geographic areas would enhance its applicability. Finally, the present study utilized a relatively small sample size, which provides an initial understanding but may have limited power to detect some effects. Larger-scale studies could build on these findings, potentially uncovering additional nuances and effects.

## Conclusions

This study provides evidence of a relationship between higher levels of experienced heterosexism, increased internalized HIV stigma, and decreased engagement along the HIV care continuum among BSMM + living in the US South. The findings highlight the significant impact of heterosexism on internalized HIV stigma and HIV care continuum engagement, illuminating the urgent need to address these intersecting challenges. As BSMM + navigate societal landscapes fraught with discrimination, it becomes increasingly vital to develop targeted strategies to mitigate the detrimental effects of internalized stigma on HIV care engagement. By identifying and implementing interventions that specifically target internalized oppression and addressing the co-occurring epidemics of discrimination and HIV, researchers and practitioners can play a pivotal role in reducing inequities for BSMM + and improving engagement along the HIV care continuum.

## Figures and Tables

**Table 1:** Characteristics of black sexual minority men living with HIV, US South (*N* = 166)

Characteristic	Total *N* = 166 *N* (%)
Mean age (SD)	30.6 (2.8)
Race*	
Exclusively Black or African American	158 (95.2)
Black or African American and another race	8 (4.8)
Ethnicity*	
Hispanic or Latinx	4 (2.5)
Not Hispanic or Latinx	158 (97.5)
Sex assigned at birth*	
Male	161 (98.8)
Other	2 (1.2)
Gender	
Man	154 (92.8)
Other	12 (7.2)
Sexual orientation	
Gay	132 (79.5)
Other	34 (20.5)
Annual income year prior	
<$19,999	54 (35.5)
$20,000 - $39,999	51 (33.6)
> $40,000	47 (30.9)
Highest level of education	
High school degree or less	67 (40.4)
Some college, Associate’s, or technical college	81 (48.8)
Bachelor’s degree or more	18 (10.8)
Employment status	
Employed full time	93 (56.4)
Employed part time	25 (15.2)
Unemployed or on disability	47 (28.5)
Health insurance	
Yes	120 (72.3)
No	41 (24.7)
Past year housing status	
Stably housed	136 (81.9)
Unstably housed	30 (18.1)

* Collected at initial Time 1 recruitment survey in 2015–2016

**Table 2:** Indirect and direct associations of experienced forms of stigma predicting HIV care continuum engagement via internalized forms of stigma (*N* = 166)

Variable b		SE	b*	*p*-value	*R*^2^ (*p*)
Direct pathways					
HIV care continuum					0.209 (0.002)
HIV care continuum on age	−0.062	0.036	−0.158	0.085	
HIV care continuum on gender	0.160	0.381	0.038	0.674	
HIV care continuum on education	0.160	0.135	0.119	0.237	
HIV care continuum on employment status: on disability	−0.488	0.512	−0.084	0.340	
HIV care continuum on employment status: parttime	−0.183	0.339	−0.061	0.590	
HIV care continuum on employment status: fulltime	0.338	0.258	0.155	0.191	
HIV care continuum on income	0.051	0.099	0.062	0.606	
HIV care continuum on experienced racism	−0.044	0.071	−0.075	0.538	
HIV care continuum on experienced heterosexism	0.023	0.076	0.038	0.765	
HIV care continuum on experienced HIV discrimination	−0.025	0.095	−0.036	0.790	
HIV care continuum on internalized racism	−0.033	0.061	−0.046	0.585	
HIV care continuum on internalized heterosexism	−0.015	0.031	−0.034	0.630	
HIV care continuum on internalized HIV stigma	−0.040	0.012	−0.259	0.001	
Internalized racism					0.244 (0.000)
Internalized racism on age	−0.015	0.044	−0.027	0.736	
Internalized racism on gender	−0.305	0.657	−0.053	0.643	
Internalized racism on education	0.061	0.154	0.033	0.692	
Internalized racism on employment status: on disability	0.225	0.601	0.028	0.709	
Internalized racism on employment status: parttime	−0.246	0.370	−0.059	0.506	
Internalized racism on employment status: fulltime	−0.146	0.283	−0.048	0.607	
Internalized racism on income	−0.242	0.118	−0.212	0.039	
Internalized racism on experienced racism	0.147	0.085	0.182	0.033	
Internalized racism on experienced heterosexism	0.017	0.113	0.020	0.857	
Internalized racism on experienced HIV discrimination	0.253	0.098	0.257	0.009	
Internalized heterosexism					0.132 (0.025)
Internalized heterosexism on age	0.038	0.082	0.042	0.643	
Internalized heterosexism on gender	−1.214	1.424	−0.126	0.394	
Internalized heterosexism on education	−0.053	0.300	−0.017	0.861	
Internalized heterosexism on employment status: on disability	−1.390	2.162	−0.104	0.520	
Internalized heterosexism on employment status: parttime	−0.458	0.695	−0.066	0.510	
Internalized heterosexism on employment status: fulltime	−0.900	0.544	−0.179	0.098	
Internalized heterosexism on income	0.114	0.203	0.060	0.574	
Internalized heterosexism on experienced racism	−0.026	0.115	−0.019	0.822	
Internalized heterosexism on experienced heterosexism	−0.072	0.173	−0.052	0.677	
Internalized heterosexism on experienced HIV discrimination	0.577	0.213	0.353	0.007	
Internalized HIV stigma					0.165 (0.007)
Internalized HIV stigma on age	0.119	0.200	0.047	0.551	
Internalized HIV stigma on gender	4.786	2.133	0.178	0.025	
Internalized HIV stigma on education	−0.802	0.726	−0.093	0.269	
Internalized HIV stigma on employment status: on disability	−0.684	3.753	−0.026	0.793	
Internalized HIV stigma on employment status: parttime	−1.952	1.921	−0.101	0.309	
Internalized HIV stigma on employment status: fulltime	−0.858	1.340	−0.061	0.522	
Internalized HIV stigma on income	0.000	0.467	0.000	1.00	
Internalized HIV stigma on experienced racism	−0.383	0.356	−0.102	0.282	
Internalized HIV stigma on experienced heterosexism	0.974	0.497	0.253	0.050	
Internalized HIV stigma on experienced HIV discrimination	0.566	0.614	0.123	0.357	
Indirect pathways					
Experienced racism → internalized racism → HIV care continuum	−0.005	0.009	−0.008	0.599	
Experienced racism → internalized heterosexism → HIV care continuum	0.000	0.002	0.001	0.858	
Experienced racism → internalized HIV stigma → HIV care continuum	0.015	0.015	0.026	0.312	
Experienced HIV discrimination → internalized racism → HIV care continuum	−0.008	0.016	−0.012	0.598	
Experienced HIV discrimination → internalized heterosexism → HIV care continuum	−0.008	0.018	−0.012	0.641	
Experienced heterosexism → internalized HIV stigma → HIV care continuum	−0.039	0.023	−0.066	0.098	
Experienced heterosexism → internalized racism → HIV care continuum	0.000	0.003	−0.001	0.900	
Experienced heterosexism → internalized heterosexism → HIV care continuum	0.001	0.004	0.002	0.775	
Experienced HIV discrimination → internalized HIV stigma → HIV care continuum	−0.023	0.026	−0.032	0.375	

*Note*. *b* = unstandardized regression coefficient. b* =standardized regression coefficient. Standardized indirect pathways approximated using the Sobel method
